# Effect of Alkali Source on Crystal Regulation and Ethanol Gas Sensing Properties of Nano-ZnO

**DOI:** 10.3390/s24237623

**Published:** 2024-11-28

**Authors:** Yinying Liao, Lu Qiu, Yunfei Ouyang, Dayang Feng, Shiyi Huang, Zhaoyang Zhang, Xinyao Xie, Junwei Ke, Tianhao Liu, Xiangxiang Chen, Hongshan Bi, Weiran Zuo

**Affiliations:** 1 Zijin School of Geology and Mining, Fuzhou University, Fuzhou 350108, China; liao_yinying@zjky.cn (Y.L.); 15805909779@163.com (L.Q.); 15170743807@163.com (Y.O.); 18185795021@163.com (D.F.); 13950791996@163.com (S.H.); zhangzhaoyang127@126.com (Z.Z.); xinyao_xie6636@163.com (X.X.); 13376998326@163.com (J.K.); thliufzu@163.com (T.L.); zuoweiran@163.com (W.Z.); 2 Zijin Mining Group Co., Longyan 364200, China; 3 Fujian Key Laboratory of Green Extraction and High-Value Utilization of New Energy Metals, Fuzhou 350108, China; 4 Department of Chemistry, University of Massachusetts, Amherst, MA 01003, USA

**Keywords:** gas sensor, ZnO, ethanoll, DFT study

## Abstract

This study investigates the ethanol gas-sensing mechanisms of ZnO nanocrystals with distinct morphologies, synthesized via a hydrothermal method using various alkali sources. Significant differences in the gas-sensing performance and morphology of ZnO samples synthesized with ammonium carbonate (Na_2_CO_3_), hexamethylenetetramine (HMTA), ammonia solution (NH_3_·H_2_O), and sodium hydroxide (NaOH) were observed. ZnO were confirmed to be impurity-free through XRD analysis, and their morphological features were characterized by SEM. TEM, XPS, and FTIR were employed to further analyze the crystal structure and binding energy of ZnO. To elucidate the underlying mechanisms, density functional theory (DFT) calculations combined with electron depletion layer theory were applied to assess charge transfer processes and identify the most sensitive ZnO crystal planes for ethanol detection. Experimental gas-sensing tests, conducted across 5–1000 ppm ethanol concentrations within a 150–350 °C range, showed that ZnO prepared with Na_2_CO_3_, HMTA, and NaOH was responsive at high ethanol concentrations as low as 100 °C, while ZnO synthesized with ammonia required 250 °C to exhibit sensitivity. All ZnO samples demonstrated excellent recovery at low concentrations at 250 °C. By integrating experimental findings with theoretical insights, this study provides a comprehensive understanding of ethanol gas-sensing mechanisms in ZnO, highlighting the role of crystal plane engineering and charge transfer dynamics as critical factors influencing gas response.

## 1. Introduction

In modern industrial processes, a range of harmful gases—including volatile organic compounds (VOCs), ethanol, formaldehyde, and ammonia—are often produced, posing risks to human health. Prolonged exposure to ethanol can seriously damage the nervous and hematopoietic systems. Furthermore, when ethanol reaches certain concentrations in the air, it can easily lead to combustion or explosion incidents [1,2]. In terms of traffic safety, drunk driving remains a leading cause of accidents. Using breath tests to detect alcohol can effectively prevent drunk driving, as these tests can be integrated with gas sensors to regulate vehicle power systems. Consequently, gas sensors are critical for environmental monitoring, industrial safety control, and the prevention of alcohol-impaired operations.

Metal oxide semiconductor (MOS)-based gas sensors have garnered widespread attention for volatile gas detection due to their high response, low production cost, and simple operational circuitry [3,4]. In recent years, MOS gas sensors have seen rapid development [5,6,7,8,9,10], with researchers focusing on micro-level modifications and tuning of MOS materials. These efforts include modulating nanoscale morphology [11,12,13,14], functionalizing with noble metals [15,16,17], and constructing heterojunctions [18,19,20] to lower the activation energy required for gas sensing reactions and to enhance response speed and response [21,22]. Among these, zinc oxide (ZnO)-based gas sensors have been widely applied for ethanol detection due to their stability, straightforward fabrication, and versatile morphologies [23]. While ZnO-based ethanol sensors have shown promising performance, the precise mechanism underlying ZnO’s response to ethanol—especially in terms of which exposed crystal faces contribute most to gas-sensing activity—remains insufficiently understood. To date, few studies have systematically examined the specific role of exposed ZnO crystal planes in ethanol sensing, nor have they quantified the relationship between charge transfer at these active sites, surface reactions, and gas-sensing performance.

In this study, ammonium carbonate (Na_2_CO_3_), hexamethylenetetramine (HMTA), ammonia solution (NH_3_·H_2_O), and sodium hydroxide (NaOH) were used as different alkali sources to control the exposure ratio of various ZnO crystal planes. Gas sensing tests revealed differences in ethanol response among the ZnO crystals, and density functional theory (DFT) calculations combined with electron depletion layer theory were employed to investigate the charge transfer mechanism, addressing a gap in the literature. By integrating experimental methods with theoretical calculations, this work provides new insights into the fundamental mechanisms of ZnO-based ethanol sensing and highlights the impact of crystal plane engineering on gas response.

## 2. Experimental Section

### 2.1. Materials and Reagents

ZnCl_2_ (zinc chloride, analytical purity), Na_2_CO_3_ (sodium carbonate, analytical purity), HMTA (hexamethylenetetramine, analytical purity), NaOH (sodium hydroxide, analytical purity), ammonia (analytical purity), and C_2_H_5_O (analytical purity). All reagents were purchased from Aladdin Reagents, Shanghai, China.

### 2.2. Synthesis of Zno by Hydrothermal Method

#### 2.2.1. Na_2_CO_3_ as a Alkaline Source for the Synthesis of ZnO Nanowires

Both 0.0015 mol ZnCl_2_ and 0.2 mol Na_2_CO_3_ were added to a beaker and dissolved in 40 mL of deionised water, and then stirred with a magnetic stirrer for 30 min at room temperature until a milky white solution was formed; the solution was then placed in a 200-mL polytetrafluoroethylene (PTFE) liner reactor, sealed and put into a blast oven, and hydrothermal reaction was carried out for 12 h at 140 °C. After the reaction is over and the reaction vessel has cooled to room temperature, the white precipitate visible in the PTFE liner is the synthesized product; the product is washed 3–4 times with deionised water and anhydrous ethanol using a vacuum filtration device to remove residual ions from the product; the washed product is placed in a blast drying oven and dried at 60 °C for 8 h. The product is recorded as ZnO-1.

#### 2.2.2. HMTA as a Alkaline Source for the Synthesis of ZnO Nanosheets

Both 0.0015 mol of ZnCl_2_ and 0.2 mol of HMTA were added to a beaker and dissolved in 40 mL of deionised water, stirred with a magnetic mixer for 30 min at room temperature until a milky solution was formed; the solution was then placed in a 200 mL PTFE-lined reaction vessel, sealed and placed in a blast oven at 80 °C for 12 h. The product was synthesised by hydrothermal reaction using a vacuum filtration device. After the reaction is completed and the reactor is cooled to room temperature, the white precipitate in the PTFE liner is the synthesis product; the product is washed 3–4 times with deionised water and anhydrous ethanol using a vacuum filtration device to remove residual ions from the product; the washed product is placed in a blast drying oven and dried at 60 °C for 8 h. The product is recorded as ZnO-2.

#### 2.2.3. Ammonia as a Alkaline Source for Synthesizing ZnO Nanoblocks

Both 0.002 mol ZnCl_2_ and 0.05 mol NH_3_H_2_O were added to a beaker and dissolved in 40 mL of deionised water, stirred with a magnetic mixer for 30 min at room temperature until a milky solution was formed; the solution was then placed in a 200 mL PTFE-lined reaction vessel, sealed and placed in a blast oven at 210 °C for 12 h. The product was synthesised by hydrothermal reaction using a vacuum filtration device. After the reaction is completed and the reactor is cooled to room temperature, the white precipitate in the PTFE liner is the synthesis product; the product is washed 3–4 times with deionised water and anhydrous ethanol using a vacuum filtration device to remove residual ions from the product; the washed product is placed in a blast drying oven and dried at 60 °C for 8 h. The product is recorded as ZnO-3.

#### 2.2.4. NaOH as a Alkaline Source for the Synthesis of ZnO

A total of 0.002 mol ZnCl_2_ was added to a beaker and dissolved in 40 mL of deionised water with 0.004 mol, 0.01 mol, and 0.016 mol NaOH, respectively, and stirred with a magnetic mixer for 30 min at room temperature until a milky solution was formed; the solution was then placed in a 200 mL PTFE-lined reactor, sealed After the reaction is completed and the reaction kettle is cooled to room temperature, the white precipitate in the PTFE liner is the synthesis product; the product is washed 3–4 times with deionised water and anhydrous ethanol using a vacuum filtration device to remove the residual ions from the product; the washed product is placed in a blast. The products were dried in a blast oven at 60 °C for 8 h to obtain the products, which were recorded as ZnO-4, ZnO-5, and ZnO-6.

### 2.3. Material Characterization

The chemical composition and crystal structure of the ZnO synthesized from different alkali sources were analyzed by XRD (BRUKER D8 Advance, Karlsruhe, Germany) patterns of different samples, and the micromorphology of the obtained samples was observed and characterized by scanning electron microscopy (SEM, Thermo Fisher Scientific, VERIOS G4, Waltham, MA, USA) detection. The micro-morphology, size and crystal structure of the samples were further analyzed by transmission electron microscopy (TEM, Thermo Fisher Scientific, Talos F200X, Waltham, MA, USA), the instrument was tested with an accelerating voltage of (200) kV. The materials were analyzed for their chemical composition and surface state by X-ray photoelectron spectroscopy (XPS, SHIMAZU, KRATOS AXIS SUPRATM, Kyoto, Japan). The chemical bonding endowment states on the surface of the obtained samples were analyzed by the potassium bromide press method on a Fourier infrared spectrometer (FTIR, BRUKER, TENSOR II, Karlsruhe, Germany), which was tested by the instrument in the wave number range of 4000–400 cm^−1^.

### 2.4. Ethanol Gas Sensing Test

**Figure 1:**
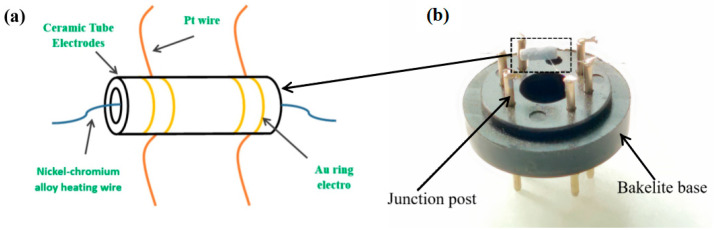
(**a**) Schematic diagram of the gas-sensing electrode; (**b**) physical diagram of the gas sensor.

The synthesized ZnO samples were prepared as gas sensor. (1) First, a certain amount of sample is mixed with a small amount of deionized water, and then put into an agate mortar for grinding and forming a slurry; (2) the slurry is carefully applied with a brush to the electrode shown in Figure 1, which is composed of a ceramic tube (length 4 mm, outer diameter 1.2 mm, inner diameter 0.8 mm), a pair of Au ring electrodes and four Pt wires. A nickel-chromium (Ni-Cr) alloy coil is inserted into the middle of the ceramic tube as a heating wire to adjust the operating temperature; (3) welding the above-mentioned elements to the binding post of bakelite base; (4) after the slurry was dried at room temperature for 30 min, the ZnO material was aged at 250 °C for 6 h.

The gas response performance of the assembled gas sensor elements was tested by using static gas distribution method on a gas sensitive test system WS-30B. The ZnO-based samples were tested in the operating temperature range of 100–350 °C. As the limitation of the experimental equipment and the test environment, and the humidity of too high and too low can affect the gas sensing performance comparation, the relative humidity of all tests is selected as a moderate humidity (40% RH). Ethanol gas was used as the target gas in this study, and the response time was defined as the time required for the sensor resistance change to reach 90% of the steady-state change after the gas was energized, and the recovery time was defined as the time required for the sensor to return to 90% of the initial resistance after the gas was expelled. Recovery time for the detection of reducing gases, the response is generally calculated as shown in Equation (1).

(1) $$S=\frac{R_{a}}{R_{g}}$$

where *S* is the response, *R_a_* is the initial resistance of the sensor in air, and *R_g_* is the resistance of the sensor when it reaches a steady state value after being exposed to target gas. During the test, ethanol is introduced into the test chamber through the injection port and is mixed with the air in the chamber under the action of a fan to reach the desired volume fraction. After the gas response has reached a steady state, the gas can be vented by opening the top cover of the test chamber.

During the whole test process, the WS-30B test system collects the voltage change value of the resistive load connected in series with the gas sensor element (the voltage of the whole test circuit is 5 V), and then the resistance change value of the gas sensor element in different gas atmospheres can be obtained through conversion. For a gas like ethanol, we can inject anhydrous ethanol (99.7%) into the built-in micro-heating plate of the gas tester to volatilize the liquid to a certain concentration. The gas distribution formula for liquid vapor is shown in Equation (2) below:

(2) $$V_{X}=\frac{V\times C\times M}{22.4\times d\times p}\times 10^{-9}\times \frac{273+T_{R}}{273+T_{B}}$$

where, $V_{X}$ is the amount of liquid injected (unit: mL), V is the volume of the test chamber (unit: mL), C is the desired gas concentration (unit: 1 × 10^−4^%), M is the molecular weight of the liquid, d is the density of the liquid (unit: g/cm^3^), P is the purity of the liquid, $T_{R}$ is the temperature at room temperature (unit: °C), and $T_{B}$ is the temperature inside the test chamber (unit: °C).

### 2.5. Computational Details

Periodic spin-polarized density functional theory (DFT) [24] was implemented using the Vienna Ab Initio Simulation Package (VASP) 5.4.1 [25] to calculate the alterations in atomistic and electronic structures of ZnO surfaces under the influence of CH_3_CH_2_OH. This analysis aimed to elucidate the characteristics of surface–adsorbate interactions. VASPKIT 1.4.0 [26] was employed for the generation of input files for VASP and for the subsequent analysis of the electronic structures obtained from VASP calculations. The simulations considered the hexagonal phase of ZnO, focusing on the (100), (110), (101), and (002) facets. Each computational box was configured to encompass a single primitive unit cell where a- and b-directions correspond to four crystal faces, and the c-direction is perpendicular to them with 20 Å of vacuum spacing between two slabs. Geometry optimizations were conducted to determine the atomic structures and energies of ZnO surfaces and isolated CH_3_CH_2_OH molecules. the calculations utilized the Revised Perdew–Burke–Ernzerhof (RPBE) exchange–correlation (XC) functional in conjunction with the DFT-D3 empirical correction for long-range van der Waals interactions [27,28,29], along with a 3 × 3 × 1 Monkhorst–Pack meshes for *K*-point sampling of the Brillouin zone and a plane-wave basis set with a cut-off energy of 500 eV.

The force convergence threshold for geometry optimization was set at 0.02 eV/Å, while the energy convergence criterion for self-consistent field (SCF) cycles was 1.0 × 10^−6^ eV. The dimensions of the computational box (a, b, and c) were optimized for ZnO surfaces and fixed at optimized values for ZnO CH_3_CH_2_OH complexes.

(3) $$\Delta G_{ads}\left({CH}_{3}CH_{2}OH\right)=G\left(MOS-{CH}_{3}CH_{2}OH\right)-G\left(MOS\right)-G\left({CH}_{3}CH_{2}OH\right)$$

where *G* (MOS-CH_3_CH_2_OH) and *G* (MOS) were contributed by electrons and vibratios

(4) $$G=\sum_{i=1}^{3N_{atoms\;}}\left\{k_{B}Tln\left[1-exp\left(-\frac{h\nu_{i}}{k_{B}T}\right)\right]+\frac{h\nu_{i}}{2}\right\}+E_{ele}+k_{B}T$$

*G* (CH_3_CH_2_OH) was contributed by electrons, vibrations, rotations, and translations:

(5) $$G=-k_{B}Tln\left[\sqrt{\frac{2\pi^{5}m^{3}}{ABC}}\frac{{\left(k_{B}T\right)}^{4}}{h^{3}P}\right]+\sum_{i=1}^{3}\left\{k_{B}Tln\left[1-exp\left(-\frac{h\nu_{i}}{k_{B}T}\right)\right]+\frac{h\nu_{i}}{2}\right\}+E_{ele}+k_{B}T$$

The Gibbs free energy formula incorporated the Boltzmann constant ($k_{B}$), system temperature (T), Planck constant (h), molecular mass of ethanol (m), and partial pressure of ethanol (P). The rotational constants (*A*, *B*, and *C*) of CH_3_CH_2_OH were derived from the optimized molecular geometry. Vibrational frequencies (νi’s) of both adsorbed and gas-phase ethanol were determined using partial Hessian analysis and atomic coordinate perturbations, with the ZnO surfaces considered static. These vibrational analyses were performed at the same computational level as the geometry.

## 3. Results and Discussion

### 3.1. Crystal Structure and Morphology Analysis of ZnO

**Figure 2:**
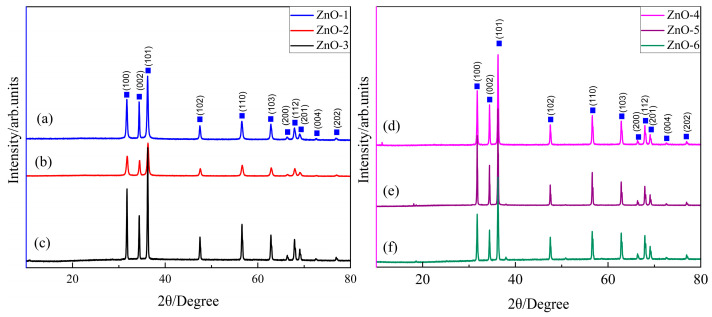
The XRD plots of ZnO. (**a**) ZnO-1; (**b**) ZnO-2; (**c**) ZnO-3; (**d**) ZnO-4; (**e**) ZnO-5; (**f**) ZnO-6.

X-ray diffraction (XRD) is the main method to study the chemical composition and crystal face distribution of samples. Figure 2 shows the XRD plots of ZnO with different morphologies prepared by hydrothermal method from different alkali sources. As shown in the figure, it can be seen from the XRD spectrum that the XRD diffraction peak can correspond to the hexagonal crystal structure of ZnO (JCPDSNo.05-0664). The eight samples have the same diffraction peak, corresponding to the (100), (002), (101), (102), (110), (103), and (200) sides of the hexagonal crystal system of ZnO at 31.751°, 34.440°, 36.252°, 47.659°, 56.763°, 63.124°, and 68.225°, respectively. The sharp and narrow diffraction peaks show that the ZnO we prepared has a good crystal structure. No peaks other than ZnO were found in the XRD diffraction pattern, indicating that the purity of ZnO we prepared was high. In addition, it can be found from the XRD diagram that the intensity and width of the XRD diffraction peak are affected by the type of alkali source, but not by the concentration of alkali source.

**Figure 3:**
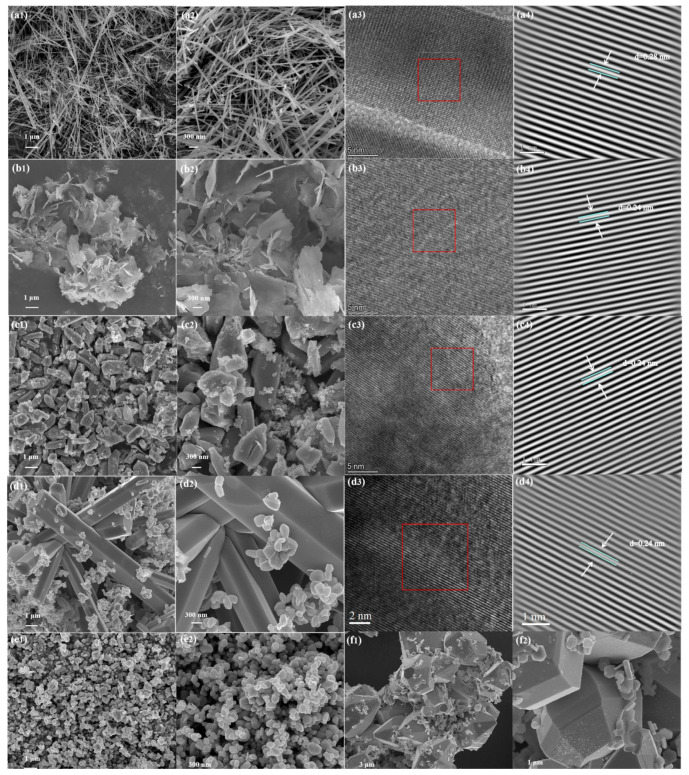
SEM and TEM characterization of ZnO samples. For ZnO-1, ZnO-2, ZnO-3, ZnO-5, ZnO-4, ZnO-6. (**a1**–**f1**) The low-magnification SEM; (**a2**–**f2**) high-magnification SEM. For ZnO-1, ZnO-2, ZnO-3, ZnO-5, (**a3**–**d3**) HRTEM; and (**a4**–**d4**) inverse FFT images from the region indicated by the solid box (red).

High resolution transmission electron microscopy (HRTEM) was used to characterize ZnO crystalline materials. By measurement, the interplanar spacing of d = 0.28 nm was measured for the facet spacing of ZnO (100) crystal faces. As can be observed from Figure 3(a1–a4), ZnO-1 prepared with Na_2_CO_3_ as the alkali source is a nanowire grown on the (100) surface with an average length of 3 μm and a diameter of 50–60 nm. As shown in Figure 3(b1,b2), the ZnO-2 sample prepared with HMTA as the base source is a three-dimensional layered nanosheet. A clear lattice fringe can be observed from Figure 3(b4) with a planar spacing of 0.24 nm, which corresponds to the ZnO (101) plane. From Figure 3(c1,c2), it can be seen that the ZnO-3 prepared from ammonia water as the base source is an irregular and coarse nanoblock, which may be due to the fact that the –OH provided by ammonia water as a base source after multi-stage hydrolysis is not stable. As can be seen from Figure 3(d1,e1,f1), the amount of NaOH has a great influence on the morphology and structure of ZnO, and when the ratio of zinc source to NaOH changes to 1:2, 1:5, and 1:8 in hydrothermal synthesis, the morphology of ZnO changes from nanoparticles growing on the (001) plane of the hexagonal crystal system to radial hexagonal nanorods and then to hexagonal nanoblocks, and it can be seen that different base sources and different base source concentrations will affect the morphology of ZnO.

**Figure 4:**
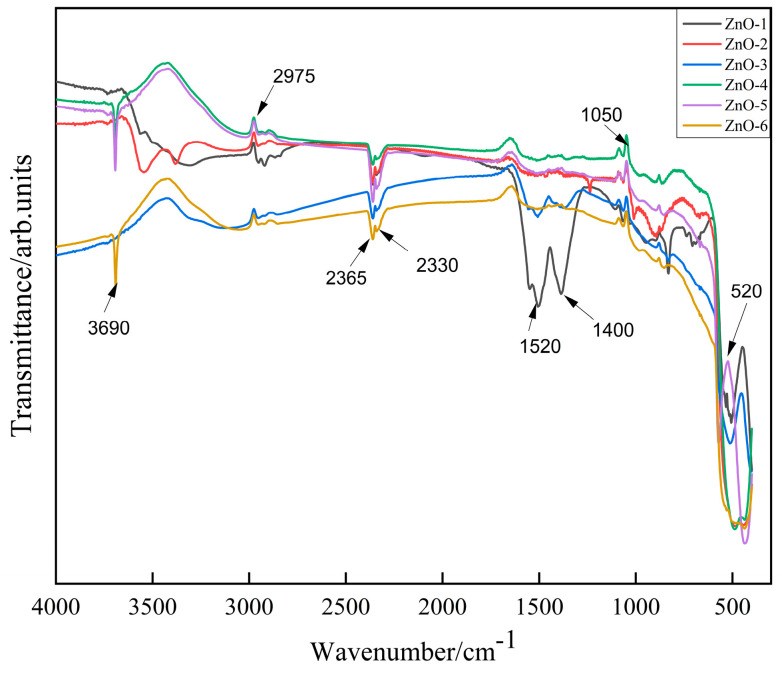
FTIR profiles of ZnO prepared from different alkali sources.

The samples were characterized by FTIR and the results are shown in Figure 4. The peak of ZnO at 520 cm^−1^ is attributed to the Zn-O stretching vibration produced by the characteristic peaks of pure ZnO. Other peaks were attributed to CO_2_ and water in the test environment, independent of the purity of the ZnO sample itself. No other bonds and groups were found in this FTIR mode, indicating that the ZnO sample was of high purity.

(6) $$D=\frac{K\lambda }{\beta \cos\theta }$$

where D is the crystal grain size, K is the scherrer constant (typically 0.9), λ is the wavelength of the X-ray radiation, β is the full width at half maximum (FWHM) of the diffraction peak, and θ is the Bragg angle.

The results calculated by the Scherrer equation are shown in Tables S5–S8 of the Supplemental Information, and the average particle sizes (ZnO-1, ZnO-2, ZnO-3, and ZnO-5) are 33.64 nm, 25.615 nm, 79.275 nm, and 261.275 nm, respectively, which are in good agreement with the SEM results.

### 3.2. Gas Sensing Characteristics

#### 3.2.1. Gas-Sensing Characteristics at Different Operating Temperatures

**Figure 5:**
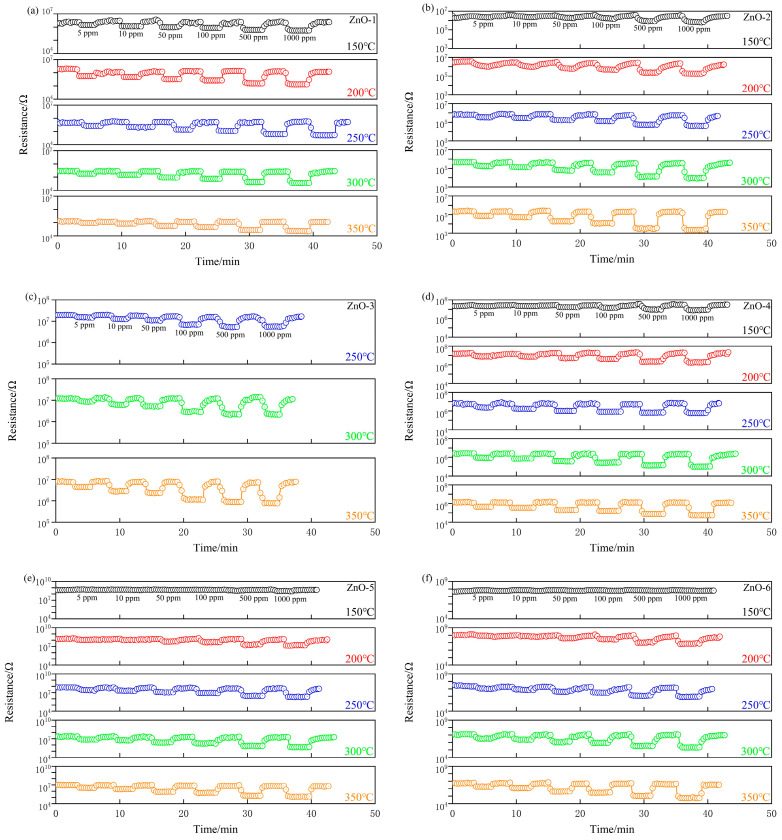
Response–recovery characteristic curves of ZnO crystals prepared from different alkali sources for ethanol gas at different temperatures. (**a**) ZnO-1; (**b**) ZnO-2; (**c**) ZnO-3; (**d**) ZnO-4; (**e**) ZnO-5; (**f**) ZnO-6.

The response–recovery characteristic curves of ZnO crystals prepared from different alkali sources for ethanol gas at different temperatures are shown in Figure 5. With the passage of ethanol gas, the resistance of the material begins to decrease, and when the ethanol gas is discharged, the resistance of the material has begun to rise, showing the n-type semiconductor response to ethanol gas, and the response of the gas sensor shows a gradual increasing trend. In addition, no significant change in the resistance of the ZnO-3 samples was observed in the range of 150–250 °C, indicating that the nano-ZnO prepared with ammonia as the alkali source has no gas-sensitive response to ethanol gas under this condition.

#### 3.2.2. Variation of Response in Response to 500 ppm Ethanol Gas at Different Operating Temperatures

**Figure 6:**
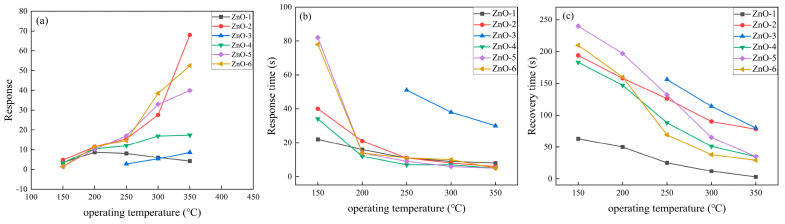
(**a**) Response; (**b**) response time; and (**c**) recovery time of ZnO crystals prepared from different alkaline sources for ethanol gas at 500 ppm at different operating temperatures.

The test temperature at which the gas sensor has the best response to ethanol gas is the optimum operating temperature of the gas-sensitive material. However, in practical production life, it is also necessary to consider the life of gas-sensitive materials and components, the response and recovery speed to ethanol gas. Due to the limitation of the instrument’s range, the response, response time, and recovery time of the ZnO-based gas sensor in the temperature range of 150–350 °C were tested in this experiment. As shown in Figure 6a, the response of ZnO-2~ZnO-6 crystals to ethanol gradually increases with the increase of operating temperature, and the maximum gas response is 68.0, 8.6, 17.4, 39.9, and 52.5 at 350 °C, respectively. The response of ZnO-1 to ethanol gas increased first and then decreased. The maximum value (8.9) is reached at 200 °C, because the surface activity of the gas-sensitive material is relatively low and the adsorption capacity of ethanol is weak when the working temperature is low, so the response is low; with the increase in temperature, the amount of adsorbed oxygen on the surface of the gas-sensitive material increases, the surface activity of the material increases, and the response increases; the desorption rate of ethanol gas is greater than the adsorption rate when the working temperature is continuously increased, and the desorption rate of ethanol gas is greater than the adsorption rate, as shown in Figure 6b,c; the response time and recovery time of all ZnO materials to ethanol decrease with the increase in temperature, which indicates that the reaction speed and desorption process of the material are accelerated at high temperature.

#### 3.2.3. Material Stability (Reproducibility) Tests

**Figure 7:**
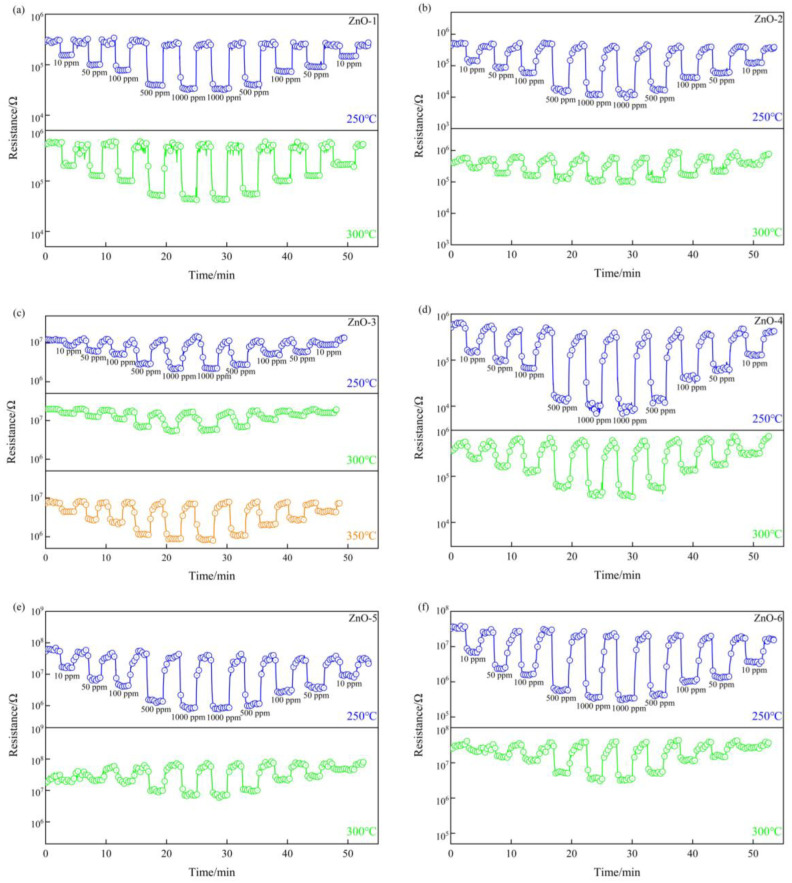
Reproducibility of ethanol gas detection by ZnO crystals prepared from different alkaline sources at different operating temperatures. (**a**) ZnO-1; (**b**) ZnO-2; (**c**) ZnO-3; (**d**) ZnO-4; (**e**) ZnO-5; (**f**) ZnO-6.

The results of the reproducibility investigation of ZnO crystals prepared from different alkali sources at different temperatures for different concentrations of ethanol gas are shown in Figure 7. As can be seen from the figure, all the samples showed good response reversibility. In each response–recovery cycle, the response time and recovery time remain basically the same, and the magnitude of the resistance change is also basically the same, indicating that the gas sensing element prepared based on ZnO crystals has good detection reproducibility for ethanol gas.

#### 3.2.4. Selectivity

**Figure 8:**
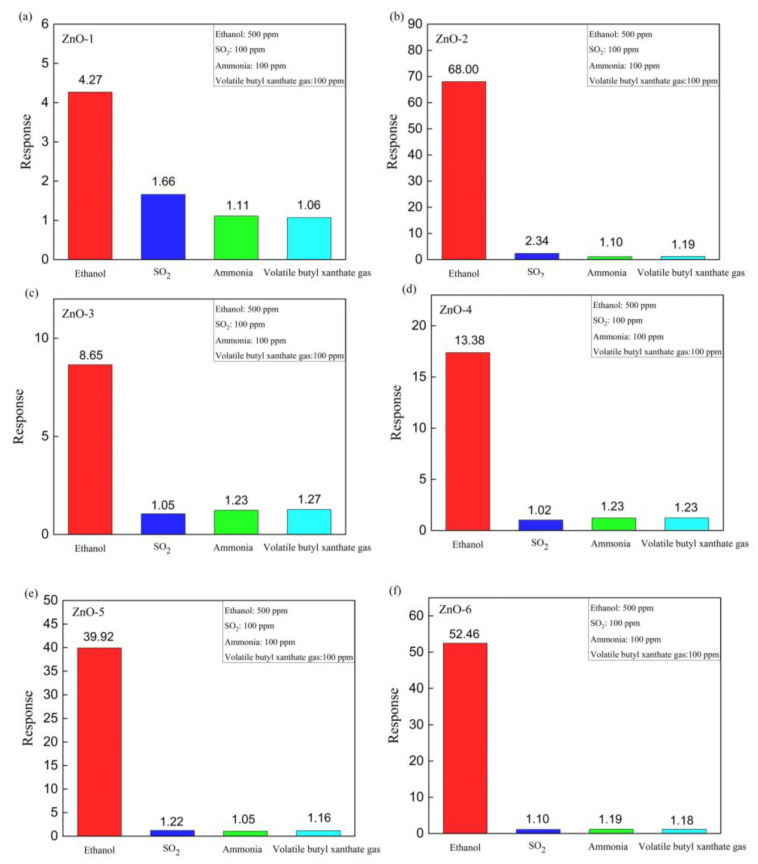
Gas sensing response of gas sensors based on (**a**) ZnO-1; (**b**) ZnO-2; (**c**) ZnO-3; (**d**) ZnO-4; (**e**) ZnO-5; (**f**) ZnO-6 at 350 °C for different gases at 100 ppm.

To further investigate the selectivity of ZnO-based sensors, as shown in Figure 8, the responses to four different gases (ethanol, SO_2_, ammonia, volatile butyl xanthate gas) were tested at an operating temperature of 350 °C, and it was found that all sensors had the highest response values to ethanol and showed excellent selectivity for ethanol.

### 3.3. Gas Sensing Mechanisms

#### 3.3.1. X-Ray Photoelectron Spectroscopy (XPS)

**Figure 9:**
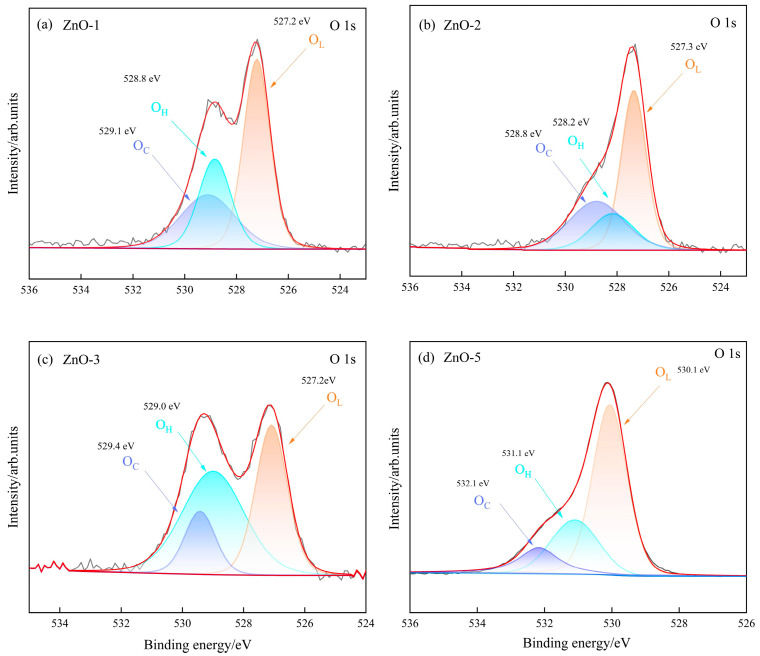
XPS spectral analysis curves of ZnO. (**a**) ZnO-1 O1s; (**b**) ZnO-2 O1s; (**c**) ZnO-3 O1s; (**d**) ZnO-5 O1s.

We chose ZnO-1, ZnO-2, ZnO-3, and ZnO-5 for XPS testing. Compared with ZnO-4 and ZnO-6, the morphology and gas response of ZnO-5 samples were in between, and the relationship between surface oxygen state and gas sensing properties of ZnO-5 materials synthesized with NaOH as base source is more representative to be studied. Figure 9a–d shows the O 1s XPS spectra of ZnO-1, ZnO-2, ZnO-3, and ZnO-5, respectively, and the O1s spectra can be decomposed into three kinds of oxygen such as lattice oxygen (O_L_), hydroxyl oxygen (O_H_), and chemisorbed oxygen (O_C_) [30]. The proportions of O_L_, O_H_ and O_C_ corresponding to the peak positions at 527.2, 528.8, and 529.1 eV for ZnO-1 were 46.37%, 26.53%, and 27.1%, respectively; the proportions of O_L_, O_H_, and O_C_ corresponding to the peak positions at 527.3, 528.2, and 528.8 eV for ZnO-2 were 48.43%, respectively, 19.04% and 32.53%, respectively; the proportions of O_L_, O_H_, and O_C_ corresponding to ZnO-3 at 527.1, 529.0, and 529.4 eV were 36.40%, 48.77%, and 14.83%, respectively; the proportions of O_L_, O_H_, and O_C_ corresponding to ZnO-5 at 530.1, 531.1, and 532.1 eV were 66.22%, 27.7%, and 5.7%, respectively, and 66.22%, 27.4%, and 11.39%, respectively.

Table S1 shows the surface oxygen distribution data obtained from Figure 9. The surface oxygen defect site of metal oxides has been confirmed in previous experiments and computational studies as the best active site for gas sensitive adsorption and charge transfer [31]. In this work, the effects of surface lattice oxygen, hydroxyl oxygen [32], and chemisorbed oxygen site ratios on ethanol gas response in XPS were discussed. As can be seen from Table S1, the increase of hydroxy-oxygen sites will occupy the originally more active lattice oxygen sites and oxygen defect reaction sites on the surface of the material, thus reducing the gas sensitive properties of the material. The calculation results in Figure S1 show that compared with surface lattice oxygen, the presence of surface chemisorbed oxygen will thermodynamically hinder the adsorption of ethanol molecules, but the surface chemisorbed oxygen begins to show adsorption activity with the increase of temperature (Figure 6a).

#### 3.3.2. DFT Calculation Results

**Figure 10:**
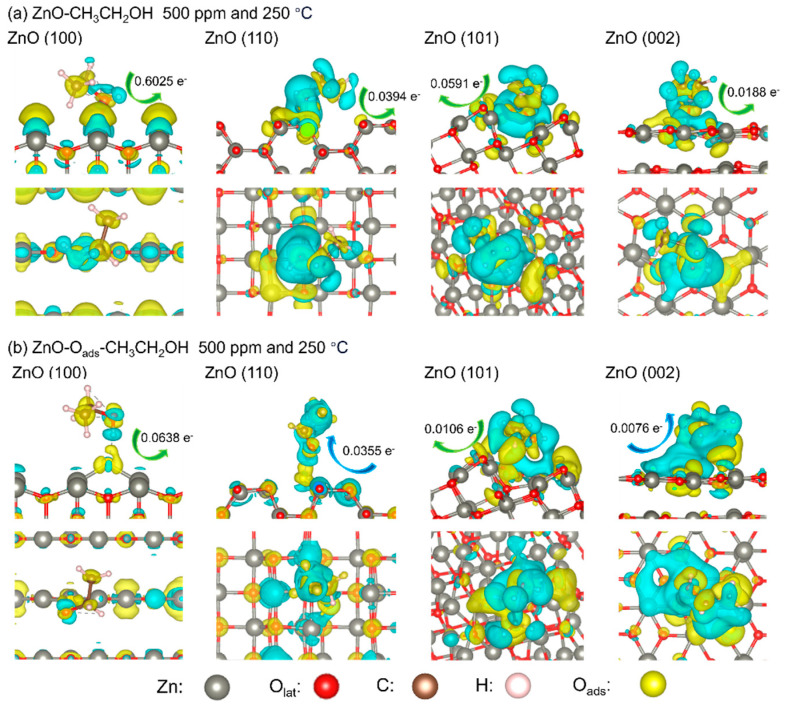
Charge transfer diagram between CH_3_CH_2_OH and representative adsorption sites on (100), (110), (101), and (002) surfaces of ZnO. The yellow and blue regions represent electron-rich and -deficient areas.

Figure 10a,b show the amount of charge transfer on the surface of the material after adsorption of ethanol molecules at the lattice oxygen and chemisorbed oxygen sites, respectively. When ethanol gas reaches the surface, ethanol molecules bond with adsorbed oxygen atoms (releasing electrons to the (100) and (101) surfaces of the material, (110) and (002) surfaces shows the electrons move to ethanol), which will cause the resistance of the material to decrease, but the amplitude of the resistance change is small and some surfaces even show side effects, resulting in a weak detection signal.

Bases on the charge transfer data from Figure 10, the proportion distribution of surface oxygen species (Table S1), and the proportion distribution of crystal surface (Table S2), we give the proportion of lattice oxygen and chemisorbed oxygen on each crystal plane and the corresponding charge transfer intensity in different materials. In the end, we present the overall surface charge transfer of ZnO during ethanol adsorption, prepared using four different alkaline sources, as shown in Table 1. The overall surface charge transfer obtained through the combination of experiment and DFT calculation is consistent with the overall trend of gas response experiment [33]. The abnormal trend between ZnO-5 and ZnO-2 is attributed to the difference in the specific surface area of the materials. It can be seen from Figure 3 that the average diameter of the nanorods of ZnO-5 is longer than that of ZnO-2. As a result, the number of active sites for ethanol adsorption in ZnO-5 per unit volume is less than that of ZnO-2.

## 4. Conclusions

This study synthesized ZnO nanocrystals with various morphologies using different alkali sources and investigated their ethanol gas-sensing properties. The results show that ZnO morphologies significantly affect gas response, with nanostructures synthesized from HMTA and NaOH exhibiting higher response, while those from ammonia and Na_2_CO_3_ responded less. The relationship between charge transfer intensity and gas-sensitive properties of different materials is given by using the DFT calculated surface charge transfer results after ethanol adsorption and the data of crystal surface proportion distribution and surface oxygen distribution in XRD and XPS, which is consistent with the experimental results. Tailoring ZnO nanomorphology offers a pathway to optimize gas-sensing performance for environmental and industrial applications.

## Figures and Tables

**Table 1:** Charge transfer and gas response of ethanol absorbed on the surface of four alkaline source synthetic materials.

Name	Total Charge Transfer	Response	Alkaline Source
ZnO-3	4.28%	8.6	Ammonia
ZnO-1	3.67%	8.9	Na_2_CO_3_
ZnO-5	6.21%	39.9	NaOH
ZnO-2	5.61%	68.0	HMTA

## Data Availability

Data will be made available on request.

## Supplementary Materials

The following supporting information can be downloaded at: https://www.mdpi.com/article/10.3390/s24237623/s1, Figure S1: (**a**) Temperature-dependent values of Δ*G*_ads_ (eV) for direct CH_3_CH_2_OH adsorptions and chemisorbed oxygen adsorbed sites at 500 ppm on (100), (110), (101) and (002) surfaces of ZnO. (**b**) Concentration-dependent values of Δ*G*_ads_ (eV) for direct CH_3_CH_2_OH adsorptions and chemisorbed oxygen adsorbed sites at 500 ppm on (100), (110), (101) and (002) surfaces of ZnO; Table S1: Three kinds of surface oxygen distribution and gas response on the surface of materials synthesized from different alkaline sources. (O_L_: Lattice oxygen sites, O_H_: hydroxy-oxygen sites, O_C_: Chemisorbed oxygen sites); Table S2: Four kinds of crystal surface XRD integral area proportion and gas response on the surface of materials synthesized from different alkaline sources; Table S3: Lattice oxygen ratio distribution on four kinds of crystal surface of materials synthesized from different alkaline sources and gas response; Table S4: Chemisorbed oxygen ratio distribution on four kinds of crystal surface of materials synthesized from different alkaline sources and gas response; Table S5: Crystal particle size of ZnO-1 samples calculated according to scherrer equation; Table S6: Crystal particle size of ZnO-2 samples calculated according to scherrer equation; Table S7: Crystal particle size of ZnO-3 samples calculated according to scherrer equation; Table S8: Crystal particle size of ZnO-5 samples calculated according to scherrer equation.
