# Supplementary material for: Effect of Alkali Source on Crystal Regulation and Ethanol Gas Sensing Properties of Nano-ZnO
Source: Sensors (Basel). 2024 Nov 28;24(23):7623. doi: 10.3390/s24237623 (PMC11644861; doi:10.3390/s24237623)
Supplement: Supplementary file 1 [file sensors-24-07623-s001.zip › sensors-3307715-supplementary.pdf]

sensors

MDPI

Support information:

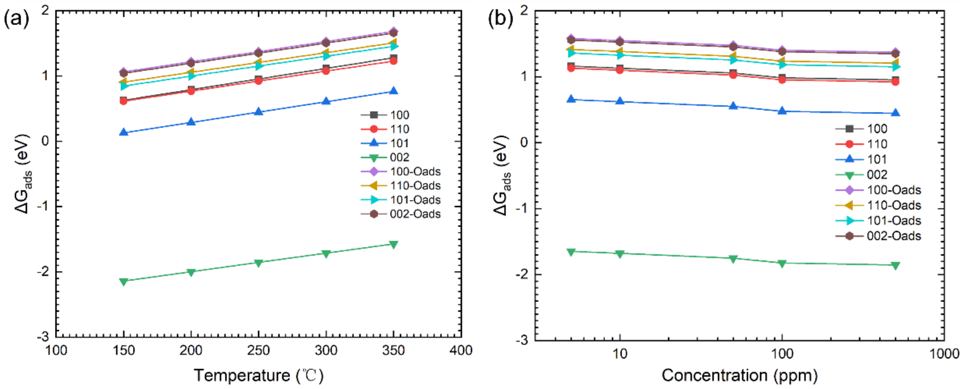

Figure S1. (**a**) Temperature-dependent values of ΔG_{ads} (eV) for direct CH_3CH_2OH adsorptions and chemisorbed oxygen adsorbed sites at 500 ppm on (100), (110), (101) and (002) surfaces of ZnO. (**b**) Concentration-dependent values of ΔG_{ads} (eV) for direct CH_3CH_2OH adsorptions and chemisorbed oxygen adsorbed sites at 500 ppm on (100), (110), (101) and (002) surfaces of ZnO.

Table S1. Three kinds of surface oxygen distribution and gas response on the surface of materials synthesized from different alkaline sources. (O_L: Lattice oxygen sites, O_H: hydroxy-oxygen sites, O_C: Chemisorbed oxygen sites)

Name	O_L	O_H	O_C	Response	Alkaline Source
ZnO−3	46.37%	26.53%	27.1%	8.6	Ammonia
ZnO−1	36.4%	48.77%	14.83%	8.9	Na_2CO_3
ZnO−5	66.22%	27.40%	11.39%	39.9	NaOH
ZnO−2	48.43%	19.04%	32.53%	68.0	HMTA

Table S2. Four kinds of crystal surface XRD integral area proportion and gas sensitivity on the surface of materials synthesized from different alkaline sources.

Name	100	002	101	110	Response	Alkaline Source
ZnO−3	11.79%	8.79%	22.01%	7.37%	8.6	Ammonia
ZnO−1	13.53%	8.48%	20.64%	6.14%	8.9	Na_2CO_3
ZnO−5	12.68%	7.50%	19.97%	7.56%	39.9	NaOH
ZnO−2	15.26%	9.25%	22.98%	7.50%	68.0	HMTA

Table S3. Lattice oxygen ratio distribution on four kinds of crystal surface of materials synthesized from different alkaline sources and gas response.

Name	100 (O_L)	002(O_L)	101(O_L)	110(O_L)	Charge transfer	Response	Alkaline Source
ZnO−3	5.47%	4.07%	10.20%	3.42%	4.11%	8.6	Ammonia
ZnO−1	4.93%	3.09%	7.51%	2.23%	3.56%	8.9	Na_2CO_3
ZnO−5	8.40%	4.97%	13.22%	5.01%	6.13%	39.9	NaOH
ZnO−2	7.39%	4.48%	11.13%	3.63%	5.34%	68.0	HMTA

Table S4. Chemisorbed oxygen ratio distribution on four kinds of crystal surface of materials synthesized from different alkaline sources and gas response.

Name	100 (O_C)	002(O_C)	101(O_C)	110(O_C)	Charge trans-fer	Response	Alkaline Source
ZnO−3	3.20%	2.38%	5.96%	2.00%	0.17%	8.6	Ammonia
ZnO−1	2.01%	1.26%	3.06%	0.91%	0.11%	8.9	Na_2CO_3
ZnO−5	1.44%	0.85%	2.27%	0.86%	0.08%	39.9	NaOH
ZnO−2	4.96%	3.01%	7.48%	2.44%	0.27%	68.0	HMTA

Table S5 Crystal particle size of ZnO-1 samples calculated according to scherrer equation.

	100	002	101	110	average
β	0.2626056	0.1838239	0.2626056	0.3413874	
2θ	31.71869	34.37101	36.222	56.54805	
D	31.46	45.24	31.46	26.4	33.64

Table S6 Crystal particle size of ZnO-2 samples calculated according to scherrer equation.

	100	002	101	110	average
β	0.3545177	0.2626063	0.341387	0.3939085	
2θ	31.78434	34.46292	36.28803	56.660058	
D	23.3	31.46	24.5	23.2	25.615

Table S7 Crystal particle size of ZnO-3 samples calculated according to scherrer equation.

	100	002	101	110	average
β	0.1578629	0.1838239	0.0474	0.210088	
2θ	31.75808	34.39727	36.23551	56.57432	
D	52.2	45.2	176.2	43.5	79.275

Table S8 Crystal particle size of ZnO-5 samples calculated according to scherrer equation.

	100	002	101	110	average
β	0.0131273	0.1575634	0.02626	0.21008	
2θ	31.77121	34.4104	36.2486	56.58745	
D	630.3	52.6	318.6	43.6	261.275
